# Health Justice as the Catalyst for Improving Health in Underserved Settings

**DOI:** 10.1093/geroni/igaa057.1859

**Published:** 2020-12-16

**Authors:** Lisa Wiese, Ishan Williams

**Affiliations:** 1 Florida Atlantic University, Boca Raton, Florida, United States; 2 University of Virginia, Charlottesville, Virginia, United States

## Abstract

Older adults face an increased incidence of Alzheimer’s disease and related dementias (ADRD) due to higher prevalence of risk factors such as lack of awareness of ADRD and reduced access to care. Furthermore, African American and Hispanic communities, which often reside in rural areas, face 1.5-2 times the risk of ADRD. The purpose of this research was to address this disparity. We conducted three studies investigating stakeholder perceptions about screening and ADRD knowledge from six diverse rural sites. Survey results revealed that regardless of rurality and education, most rural (71%) older adults believed heart disease and poor diet increased ADRD risk and were willing to be screened. Yet only 15% had been tested for memory loss by their provider. Rurality was the only significant predictor of willingness to participate in screening. It is important to maintain a focus on pursuing health justice among rural older adults facing multiple health disparities.

